# Case report: inflammatory pseudotumor in the lung parenchyma caused by a medical suture originating from a cardiac surgery 35 years ago

**DOI:** 10.1186/s13019-020-01194-z

**Published:** 2020-06-23

**Authors:** Shushi Meng, Ganwei Liu, Shaodong Wang, Fan Yang

**Affiliations:** Department of Thoracic Surgery, Peking University People’s Hospital, No. 11 Xizhimen South Street, Beijing, 100044 China

**Keywords:** Inflammatory pseudotumor, Iatrogenic foreign body, Medical suture

## Abstract

**Background:**

The incidence of the iatrogenic foreign body retained after surgery is extremely low. Iatrogenic foreign body retained is surrounded by normal tissue, which responds to foreign matter to form inflammatory pseudotumors. Surgical sponge or swap is the most common type of foreign body. There were no reports of medical sutures remaining as foreign bodies in the lung parenchyma to form inflammatory pseudotumors.

**Case presentation:**

A CT scan of a 50-year-old female showed an irregular soft tissue mass in the left upper lobe with rough edge and spiculation. After 20 months, the size increased from 2.8 × 1.9 cm to 3.2 × 2.2 cm. The patient underwent a ventricular septal repair surgery for congenital Fallot tetralogy 35 years ago and a left breast surgery for breast cancer. She had a family history of lung cancer. Evaluation of this mass highly suggested a lung malignant lesion. The patient underwent video-assisted thoracoscopic surgery (VATS) lobectomy and her pathology revealed an intrapulmonary inflammatory pseudotumor caused by a medical prolene suture. Based on her medical history and other reports of iatrogenic foreign bodies, we believe that this suture retained from the heart surgery 35 years ago entered the pulmonary artery, moved to the distal branch, and eventually formed an inflammatory pseudotumor in the lung parenchyma. Here we reported and analyze this rare case.

**Conclusion:**

We reported a rare case of inflammatory pseudotumor in the lung parenchyma caused by a medical suture, and determined it was a prolene suture retained in the body during a cardiac surgery 35 years ago. Diagnosis of this rare disease required sufficient imaging experience. Besides, appropriate surgical exploration can help with the diagnosis and treatment.

## Background

The iatrogenic foreign body retained after surgery is a serious medical fault, and its incidence is extremely low under the current strict and standardized medical procedures. Iatrogenic foreign body retained is surrounded by normal tissue, which responds to foreign matter to form inflammatory pseudotumors. Surgical sponge or swap is the most common type of foreign body. There were no reports of medical sutures remaining as foreign bodies in the lung parenchyma to form inflammatory pseudotumors.

## Case presentation

A 50-year-old woman presented with a lesion in the left lung for 20 months on routine examination. Computed tomography (CT) at a local hospital showed that there was an irregular mass in the upper lobe of her left lung, about 2.8 × 1.9 cm in size, with a clear boundary, peripheral spiculation, and stretched adjacent pleura. The patient had no symptoms such as cough, sputum, dyspnea, fever, and hemoptysis. One month ago, she underwent surgery for the left breast cancer in the local hospital. A second chest CT showed no significant changes in the mass. Positron emission tomography-CT (PET-CT) revealed an irregular high-density mass in the left lung with increased fluorodeoxyglucose (FDG) uptake, no significant abnormal FDG metabolism in the rest of the body, multiple small lymph nodes in the left axilla, and slightly increased FDG uptake in some lymph nodes.

**Fig. 1:**
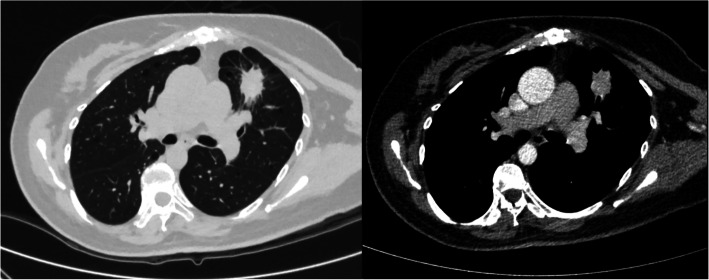
Chest CT at our hospital showed an irregular soft tissue mass in the anterior segment of the upper lobe of the left lung

For further treatment, the patient was admitted to our department. There was no obvious abnormality on physical examination. The chest CT showed an irregular soft tissue mass in the anterior segment of the upper lobe of the left lung, and the border was not clear. The cross-sectional area was about 3.2 × 2.2 cm, and the spiculation sign could be seen. The plain CT value was 30 HU. Calcification of the patch was obvious after enhanced scanning, and the CT value was 98 HU. The mediastinal structure was clear, and no obvious enlarged lymph nodes were seen (Fig. 1). There were no significant abnormalities in blood sample tests, including tumor markers. The patient had suffered from tetralogy of Fallot and underwent surgery for a ventricular septal repair35 years ago. Breast-conserving surgery was performed for her left breast cancer 1 month ago, and the pathological report confirmed a lobular carcinoma in situ. She had a family history of lung cancer (grandfather) and no smoking history.

According to the patient’s medical history and imaging findings, this mass was highly suspected to be malignant. The patient underwent video-assisted thoracic surgery (VATS) instantly without preoperative CT-guided percutaneous needle lung biopsy. The intraoperative palpation of the lesion suggested that the lesion was too close to the hilum to receive limited resection. So we performed the lobectomy of left upper lobe directly. The procedure was successful and the mass was completely removed.

**Fig. 2:**
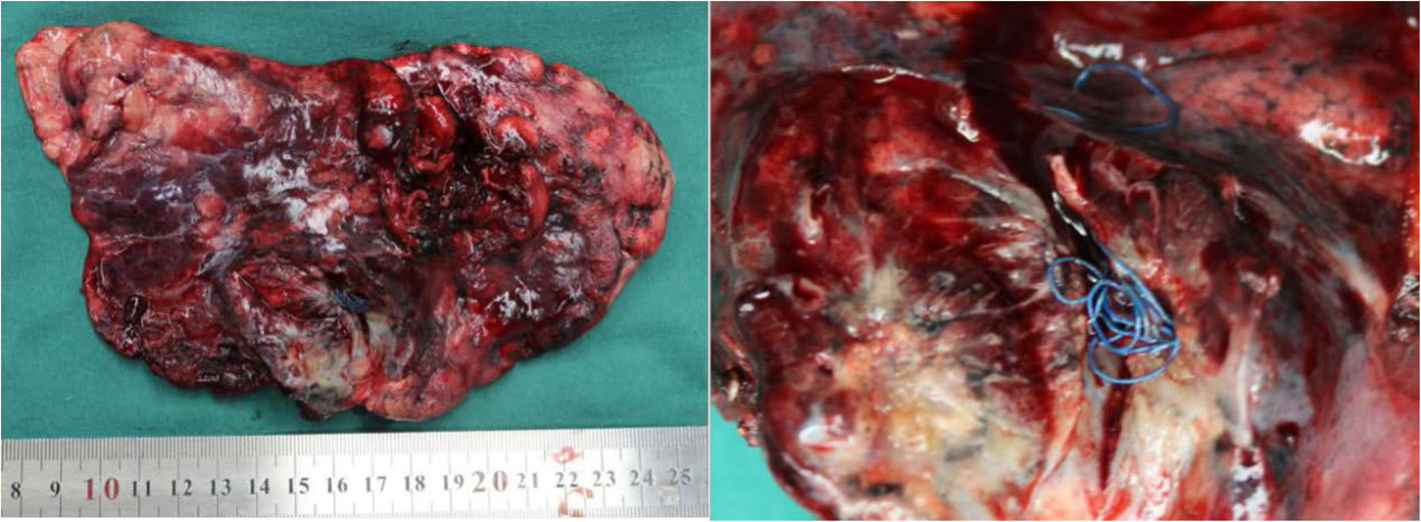
Surgical specimen (left: the left upper lobe, right: a blue medical suture was visible inside)

The dissected mass was gray-yellow, and a blue medical suture was visible inside (Fig. 2). The surrounding tissue showed inflammatory changes, and local necrosis was found. The suture is about 15 cm in total length. The pathology report suggested that the mass tended to be an inflammatory pseudotumor, and no malignant cell component was seen. The structure of the lung tissue in the lesion area was damaged. The proliferative vascular components were visible, the wall thickness was accompanied by hyaloid and mucus degeneration, and fibrous tissue hyperplasia with hyaloid. Lymphocytes, plasma cells, and neutrophils infiltrated in the lesion area, and the remaining alveolar epithelium showed reactive hyperplasia. The submitted lymph nodes also showed reactive hyperplasia. Immunohistochemical staining results: CD38 (+), IgG (+), IgG4 (focal +), IgG4 / IgG less than 40% +. Special staining results: silver hexamine (−), Congo red (−).

The patient recovered well and discharged on the 3rd day after operation. Follow-up after 3 months showed no special abnormalities.

## Discussion and conclusions

In this case, the surgical specimen showed a mass of medical sutures and inflammatory changes in the surrounding lung tissue. The pathological diagnosis was inflammatory pseudotumor caused by iatrogenic foreign body. It is recognized by surgeons that this suture is a blue prolene suture, 2–0 size, which is often used in cardiovascular surgery. According to the patient’s history of ventricular septal repair surgery, we fully believe that this iatrogenic foreign body is a medical suture left over from the cardiac surgery performed 35 years ago, and eventually formed an inflammatory pseudotumor in the lung parenchyma.

It is very rare for medical sutures to remain in the lung parenchyma as iatrogenic foreign bodies. Cases of medical foreign bodies such as medical guidewires, catheter fragments, embolization coils, and venous filters remaining and moving in large blood vessels have been reported [1]. Therefore, we can speculate that the suture left during a cardiac surgery entered the pulmonary artery and gradually moved to the end of the branch, and eventually an inflammatory reaction occurred in the lung parenchyma, forming an inflammatory pseudotumor.

A retrospective survey showed that of the 191,168 operations performed in Mayo Clinic, Rochester (MCR) from 2003 to 2006, 34 cases of retained iatrogenic foreign bodies occurred (0.178 / 1000). Items retained included 23sponges (68%), 7 miscellaneous items (20%), 3 needles (9%), and 1 instrument (3%) [2]. Iatrogenic foreign body is often surrounded by normal tissue, which responds to foreign matter to form inflammatory pseudotumors. This lesion is often called gossypiboma because surgical sponge or swap is the most common type of foreign body. The term “gossypiboma” originates from the Latin gossypium, referring to cotton wool [3].

According to a systematic literature review of 254 case studies, gossypiboma occurs mainly in the abdomen (56%), followed by pelvis (18%) and thorax (11%) [4]. Patients mostly complain of pain, palpable mass, and persistent fever; but there are also very few patients without any discomfort but only abnormal imaging findings. The most common detection methods used are CT (61%), radiography (35%), and ultrasound (34%) [4]. It is generally believed that magnetic resonance imaging and biopsy can also assist the diagnosis, but false negatives of biopsy pathological results cannot be ignored.

Iatrogenic inflammatory pseudotumors mainly manifest as solid tumors with smooth borders [2, 5]. The preoperative CT in our case showed irregular lesion shape, rough border, pleural traction sign, and gradual enlargement, which could not rule out manifestations of a malignant mass. The patient had a history of congenital heart disease, and breast cancer, and a family history of lung cancer. Therefore, malignant tumor in the lung need to be considered, and Surgical exploration is necessary. VATS surgical exploration is less harmful to patients. Considering the large size of the lesion, a lobectomy was performed. Like most reported cases, our patient had a good prognosis after removal of the lesion.

We report a rare case of inflammatory pseudotumor in the lung parenchyma caused by a medical suture. Based on the patient’s medical history, imaging data, and specimen, we determined that this suture was a prolene suture left in the body during a cardiac surgery performed 35 years ago. Detailed history tracing and accumulation of imaging identification are helpful in the clinical diagnosis of an iatrogenic inflammatory pseudotumor. Once it occurs, appropriate surgical exploration can help with the diagnosis and treatment.

## Data Availability

Not Applicable.

## Abbreviations

CT: Computed tomography
VATS: Video-assisted thoracoscopic surgery
PET-CT: Positron emission tomography-CT
FDG: Fluorodeoxyglucose
MCR: Mayo clinic, Rochester
